# Strategic Shift to a Diagnostic Model of Care in a Multi-Site Group Dental Practice

**DOI:** 10.16966/2378-7090.209

**Published:** 2016-06-20

**Authors:** E Kalenderian, P Maramaldi, S Kim, J Etolue, L McClellan, K Simmons, A Yansane, JM White, MF Walji, RB Ramoni

**Affiliations:** 1 Department of Oral Health Policy and Epidemiology, Harvard School of Dental Medicine, Boston, MA, USA; 2 Simmons School of Social Work, Boston, MA, USA; 3 Willamette Dental Group, Hillsboro, OR, USA; 4 Department of Preventive and Restorative Dental Sciences, School of Dentistry, University of California, San Francisco, USA; 5 Department of Diagnostic and Biomedical Sciences, The University of Texas Health Science Center at Houston, School of Dentistry, Houston, Texas, USA; 6 Center for Biomedical Informatics, Harvard Medical School, Boston, MA, USA

**Keywords:** Diagnosis, Policies, Forms, Dentistry, Vision, Leadership, Framework

## Abstract

**Background:**

Documenting standardized dental diagnostic terms represents an emerging change for how dentistry is practiced. We focused on a mid-sized dental group practice as it shifted to a policy of documenting patients’ diagnoses using standardized terms in the electronic health record.

**Methods:**

Kotter’s change framework was translated into interview questions posed to the senior leadership in a mid-size dental group practice. In addition, quantitative content analyses were conducted on the written policies and forms before and after the implementation of standardized diagnosis documentation to assess the extent to which the forms and policies reflected the shift. Three reviewers analyzed the data individually and reached consensuses where needed.

**Results:**

Kotter’s guiding change framework explained the steps taken to 97 percent utilization rate of the Electronic Health Record and Dental Diagnostic Code. Of the 96 documents included in the forms and policy analysis, 31 documents were officially updated but only two added a diagnostic element.

**Conclusion:**

Change strategies established in the business literature hold utility for dental practices seeking diagnosis-centered care.

**Practical Implications:**

A practice that shifts to a diagnosis-driven care philosophy would be best served by ensuring that the change process follows a leadership framework that is calibrated to the organization’s culture.

## Introduction

The dental profession is on the cusp of a transformative change: documenting dental diagnoses using standardized terms. IHTSDO, the International Health Terminology Standards Development Organization, which owns the license for SNOMED-CT [1], the most complete reference terminology for all medical and dental terms, has formed a dental Special Interest Group (SIG) and has in its 2015 work plan specifically identified working with “the Dental SIG providing a content review and gap analysis of dental content” [2]. Moreover, IHTSDO notes that it is doing the work with the dental SIG “as part of the agreement with the American Dental Association (ADA)” [2]. Leading this evolution, an inter professional research group of dental and health researchers representing four institutions has developed a comprehensive dental diagnostic interface terminology, the DDS terminology (formerly named the EZCodes) [3]. This has sparked a renewed focus on the development, implementation, dissemination and research of the use of standardized diagnostic terms and codes, especially for the electronic health record (EHR) in the dental community recently [4–8].

In fact, the change may be seen as something that goes beyond simple documentation to a shift from a treatment-centered approach to care to one that is diagnostic-centric. Dentistry’s historical focus on treatment has been reinforced by the fact that it is not mandatory to document the diagnosis as part of the billing process: dental billing is based upon procedure codes, the CDTs [9]. Diagnosis should be the primary aim of the dentist, as proper diagnosis is a prerequisite to proper treatment. This is underscored by recent findings in the medical realm, in which an analysis of the National Practitioner Data Bank identified that diagnostic errors are the most common basis for malpractice claims and are the most dangerous of medical mistakes [10].

How can the dental profession navigate this change most effectively? The reality is that the change will not happen at the academic level: it will succeed or fail within practices. Change is inevitable aspect of a professions evolution. So too is resistance to change. In Leading Change, Kotter describes the eight critical steps necessary to assure success when guiding changes (see column 1 table 1) [11].

A privately held, accountable-risk bearing entity that provides individualized dental treatment programs for over 400,000 members with over 50 dental practices in the Pacific Northwest recently committed to implementing DDS standardized diagnostic terms as part of their transition to an electronic health record. Within a short period of time, they achieved a remarkable utilization rate of 97%. Determination of the utilization rate has been reported elsewhere [12]. This gave us the unique opportunity to better understand whether and how the practice used documented forms and policies to reinforce the transition to mandatory standardized diagnostic term documentation. Specifically, we wanted to understand the context in which these diagnostic terms were used within their revised documentation and what impact the changes may have had on the organization. Most importantly, the remarkable success rate in adoption of the DDS terminology and thus becoming diagnostic centric, for this group practice was analyzed and within the context of the eight critical steps outlined for change guidance.

## Purpose of Study

The purpose of this study was to understand what actions the leadership of a large privately held, accountable-risk bearing entity serving over 400,000 member stook in order to succeed in the implementation of an EHR and Dental Diagnostic Terminology. Kotter’s framework [11] was used to analyze the successful shift to diagnostic centrality in a relatively short period of time. The analysis is intended to provide guidance to other organizations wanting to replicate or amend this approach. Secondly, we tried to understand to what extent forms and written policies reflected a diagnostic-centric philosophy, both before and after the group practice’s shift to structured documentation of dental diagnoses in an EHR.

## Methods

### Context

The study was conducted at a privately held, accountable-risk bearing entity that provides individualized dental treatment programs for over 400,000 members with over 50 offices in the Pacific Northwest. The offices range in size from 1–10 dental providers with a range of 4–37 staff to support the providers depending upon the size of the office. Each office has a managing doctor responsible for the day-to-day management of operations. The overall direction for the organization is however set by the CEO, COO, and an organizational structure of regional directors assures implementation of and adherence to the organization’s vision. The practice implemented the DDS dental diagnostic interface terminology in conjunction with its conversion to an EHR in November 2012. Permission for the study was obtained from the dental group practices leadership, and IRB approval (protocol #23901) for the study was obtained from the Harvard Medical School’s Institutional Review Board.

### Discovering the leadership steps to develop diagnostic centrality

We translated Kotter’s guiding change framework [11] into eight interview questions focused on the simultaneous adoption of the EHR and the diagnostic coding within the practice group. A trained member of the research team interviewed ten senior managers, using a semi-structured interview approach. Table 1 [11] summarizes the findings.

### Document identification

We reviewed all forms (e.g., referral form, triage form) and policy documents (e.g., protocols and procedures) at the practice both prior to (December 2011) and after (January 2014) the implementation of the DDS terminology. Of the total of 101 documents, five were excluded: the reasons for exclusion were that they were either patient-focused or purely graphical (e.g., an odontogram). Table 2 shows the distribution of the reviewed documents. In total, eighteen forms and 78 policies were included in the analysis.

### Document content analysis

We used a quantitative content analysis approach in which the complete content of each document was manually reviewed. We analyzed the content of each document with respect to three elements: 1) presence of the word “diagnosis” or any variation of it (diagnose, diagnosed, diagnostic, dx, diagnoses); 2) presence of a description of a diagnosis (e.g., impacted teeth, abscess formation); and 3) presence of references to specific diagnostic codes (e.g., DDS 346218 as DDS code#, 520.4 as ICD 9 code #, K00.4 as ICD 10 code#, or 80289005 as SNOMED CT code#).

We captured the data both in terms of (1) yes/no presence of one or more instances of each element in a given document and (2) the frequency of the elements. Three reviewers participated in the process: one researcher performed the task of extracting the data. Two additional researchers then reviewed the results and the group met to reach consensus where there were differences.

## Results

### Interview with senior managers

The CEO and COO were the driving forces behind the initial changes. They established the sense of urgency, drafted the vision, and forced a guiding coalition of leaders through out the practice group and management team. They communicated the vision effectively using existing venues and an extensive training program that included the unprecedented step of suspending routine clinical and administrative services in each of the 53 offices in order to transport every eligible employee to a central training location. In some cases, temporary staff was utilized when most of the permanent staff in a smaller clinic participated in the training session on the EHR and diagnostic codes, in order to be available for emergency care.

Broad-based action was accomplished by identifying and eliminating barriers using PDSA cycles and identifying 1 to 2 EHR experts known as “super users” at each of the 53 practice locations. The super users provided ongoing on-site training. Short-term wins were celebrated through personal emails and calls by the CEO as well as office lunches and gift cards to local coffee shops when metrics were accomplished. The standardized documentation of diagnostic codes in the EHR allowed the organization to develop metrics for success, ensuring adherence to the practice mission of providing primary preventive care.

Evidence of a lasting culture is seen in the fact that the CEO no longer has to champion diagnostic centric-care, as providers at all locations promote the vision to new hires and patients voluntarily.

### Documentation review

Fifty-one pre-implementation documents and 45 post-implementation documents were reviewed, for a total of 96 documents. Of the 45 post-implementation documents reviewed, 39 post-implementation versions were exactly the same as the pre-implementation versions we reviewed earlier. Of these 45 documents, 31 were officially updated in the one-year since the former review date. Most documents were reviewed and just received an updated date stamp; 6 documents had their content revised, but only two of the policies added a diagnostic concept. Thus the updating effect with regard to the word “diagnosis” or “description of a diagnosis” was 6.7% for the policies and 6.5% for all documents together (Table 3).

None of the forms or policies contained a diagnostic code, either in the post-implementation or pre-implementation version. The word “diagnosis” and its variants appeared more often in policies than in forms (39% vs. 14% for the respective post-implementation versions). Descriptions of diagnoses were most frequent in policies (71% in post-implementation) and are appearing less in forms (57% post-implementation forms vs. 64% pre-implementation forms) (Table 4).

We conclude that documentation changes were made as a process of routine updates.

## Discussion

Cultivating a diagnostic centric care model through the use of a dental diagnostic terminology is a change in the modus operandi for the dental profession and in increasing numbers, dental practices. We consider the implementation of a dental diagnostic terminology an innovation, in the sense that it is “the introduction of something new (an idea, method or device)” [12]. More explicitly, an innovation can be defined as a novel set of behaviors, routines and ways of working, which are directed at improving health outcomes, administrative efficiency, cost-effectiveness, or user experience, which are implemented by means of planned and coordinated action [13]. It requires dedication, training and vigilance to assure the diagnostic terminology is used consistently and correctly [12].

Kotter [11] posits that communication is fundamental to leading successful change. The practice group that we studied achieved a 97 percent utilization rate, despite the fact that few documents were updated to reflect the shift to diagnostic-centric care. Our findings indicate that the practice’s success may be due to following many of the steps in the model exquisitely well. A clear sense of urgency was created by the CEO, who together with the COO leveraged a specific date as the deadline (“big bang”), when the entire organization - 53 offices in three states - was switched from paper to the EHR including diagnostic codes. A powerful coalition was formed among providers and support staff who believed in the vision and mission “to provide the best possible care to patients.” Additionally, training was strategically executed throughout the organization, and presented as an incentive and reward for commitment to the mission. Short-term wins and additional change were created through the ongoing development of sophisticated feedback mechanisms, including personal emails and phone calls from the CEO, office lunches, and compensation incentives. When the management was assured that the providers felt confident in the use of the EHR and diagnostic codes, the practice un-blinded the data to create healthy competition that created further incentives to perfect the use of diagnostic codes.

Although the practice did not immediately update its policies and procedures, it has since done so, which may be a vital part of sustaining change in this organization. According to Kotter, most change comes 36 months after the initial wins [11]. The change will stick when it becomes part of the culture. Ensuring that all relevant policies and procedures are updated and implemented to reflect the new vision is one way to facilitate that the new vision is rooted in the social norms and shared values of the organization.

Understanding the culture of the organization is important to any change effort. In order to understand the organizational culture of the multi-specialty group dental practice described in this study we used a framework developed by Handy [14] which describes four archetypes:

- **Power culture:** Power is concentrated at a central source, such as an owner or President. Minimal bureaucracy exists, and staff functions with few rules, policies and procedures.
- **Role culture:** A bureaucratic culture in which each unit is a pillar supporting the organization. Policies and procedures control the organization, and employees operate based on job descriptions.
- **Task culture:** A dynamic culture in which activity typically occurs in the context of groups formed to accomplish specific goals, which disband when the task is complete.
- **Person culture:** This culture is typified by consensus decision-making and exists to serve and support the individuals within it.

The practice reported here has both a power culture and role culture. The CEO was the centralized power that initially developed and launched the vision and change. At the same time, the 53 practices function assemi-autonomous pillars on a day-to-day basis within the boundaries of the centrally developed policies and procedures. The influential role of the CEO supported by the observation that, despite the fact that practice documents did not explicitly reflect the shift to a diagnostic-centric approach, the practice has achieved an impressive DDS terminology utilization rate. A “role culture” organization would have required a more complete institutionalization of the change in documents. Furthermore, some researchers posit that implementation is fastest in a top-bottom (top-down) organizational structure, partly because end users may resist adoption until prompted by their managers [13,15].

Process also serves to support innovation. The dental diagnostic interface terminology was fully implemented by the group practice simultaneously with the conversion from paper records to electronic health records. Company-wide policy made the use of the DDS dental diagnostic terminology in the EHR mandatory and the EHR was enriched with a clinical decision support feature informing providers when a dental diagnostic term was not used. This feature forced dentists to use the dental diagnostic terminology. The EHR was programmed to require a diagnostic term to be entered in the record. A diagnostic term was required before the provider could proceed with the case, and to close a patient’s electronic patient’s chart. As a result, the structure and function of the EHR assured compliance and set a standard of care.

Consistency is another important managerial tool to ensure alignment within the organization and to sustain change after initial wins [16]. Policies and procedures reflected the new diagnostic centric approach facilitates communication of the new vision throughout the organization.

Managers of practice groups planning the implementation of change, such as dental diagnostic codes, must calibrate policies and procedures to reflect its new vision. The ideal approach according to Kotter, is to use all communication channels to express the vision [11]. As a first step, scanning all forms and policies will be informative to see how diagnostic centric the organization is “on paper” and will reveal what opportunities exist to support the vision. The impact of these changes will depend upon the organizational culture in which the change is taking place.

## Conclusion

As dental practices move towards becoming diagnosis-centered, established change frame works may increase probability of success. Secondly, updating documented policies and forms to reflect that philosophy should be seen as part of the implementation process. While updated policies and procedures function as an important means of communicating a change in practice, more importantly, they may be a vital part of sustaining change in certain organizational cultures.

## Figures and Tables

**Table 1:** Findings from senior managers’ semi-structured interviews using Kotter’s guiding change framework [11]

**1. Establish a sense of urgency** Examining the market and competitive realities Identifying crises, potential crises or major opportunities
Practice Group (PG) leadership had a decades-long philosophy to provide evidence-based treatment similar to a primary care medical model. As one put it, “in primary (care) medicine it would be perceived as almost criminal to proceed with a procedure without a diagnosis.” While previous attempts to develop or modify software programs designed to monitor service delivery using paper records and limited diagnostic codes had failed, competitors were perceived to be moving ahead with the adoption of EHRs. The comprehensive DDS codes combined with the successful implementation of the EHR would allow for total utilization review (review of services provided, specialty referrals and authorization, high-cost case management, and billing audit) rather than small samples that had traditionally been limited by inconsistent diagnostic codes and paper records.
**2. Form a powerful guiding coalition** Putting together a group with enough power to lead the charge Getting the group to work together like a team
One year before actual implementation, the CEO and COO worked in tandem to explain the benefits of the adoption of diagnostic codes and full implementation of the EHR to both senior clinical and operations managers. With the unanimous support of senior management, a core implementation team was strategically appointed to include representatives from all employee constituencies within the organization. It included early adopters who were enthusiastic, as well as known stragglers who wanted more proof before buying in. Once there was buy in, the operations team charged with implementation was positioned to shift the entire organization, at all levels, to the EHR and diagnostic terms. As one respondent put it, the twelve member “ops” team “included all walks of life” within the practice group.
**3. Develop a vision and a strategy** Creating a vision to help create the change effort Developing strategies for achieving that vision
As one senior manager explained “It was urgent for us … to practice our promise to our customers and our patients, to follow our mission statement.” The practice group promotes a proactive, preventive evidence based model. As one put it, “physician of the mouth first, technician second.” The diagnostic codes in the EHR allowed the PG to assure that the treatment is appropriate. The general perception was best captured by one comment that the PG has a “legal, ethical, and moral obligation” to patients to be sure that the oral environment is conducive to the procedure.
**4. Communicate that change vision** Using every vehicle possible to communicate the new vision and strategy Having a guiding coalition role model, messaging what is expected of employees
After the CEO, COO, and senior management group agreed to pursue the EHR and diagnostic codes, they planned for regular and comprehensive communication through every layer of the practice group. The guiding coalition started with CEO, COO, and senior management messaging the importance of the EHR and diagnostic codes. The CEO and COO attended and communicated the vision through several existing venues that included each of the 53 offices across three states. This included quarterly management meetings (every managing doctor and office manager); quarterly managing doctor meetings; quarterly doctor meetings (mandatory for all dentists); quarterly managing dentists meeting (all either in-person or remotely. During the training period, the PG invested approximately $2 million to transport, house, and feed every employee during centralized training.
**5. Empowering broad-based action** Getting rid of obstacles Changing the systems or structures that undermine the change vision Encouraging risk-taking and non-traditional ideas, activities, and actions
The ops team was lead by the project manager who was sanctioned and supported by the entire senior management including the CEO and the COO. The ops team used Plan, Do, Study, Act (PDSA) cycles to identify obstacles and to find solutions. Each of the 53 sites was required to submit a report identifying problems (obstacles) and to suggest systems changes that would aid in the implementation of the diagnostic codes and EHR. The ops team identified subject matter experts in areas related to EHR implementation. Additionally, the ops team identified one or two “super users” in each of the 53 office locations. The super users in each office provided technical assistance to those who were having trouble using the EHR. It was not unusual for senior doctors to welcome support from super users (often dental assistants) on how to use the EHR.
**6. Generating short-term wins** Planning for visible improvements in performance or wins Creating those wins Visibly recognizing and rewarding the people who made the wins possible
The entire EHR system was implemented in a single day, which came to be known as the “BIG Bang.” The ops team, subject matter experts, and super users, through the use of PDSA cycles, identified wins and transmitted them throughout the organization. When individuals and offices made gains, they were recognized by announcements, personal emails and phone calls from the CEO and COO, pizza parties and/or gift cards from local coffee shops.
**7. Consolidate gains and producing more change** Using increased credibility to change all systems, structures and policies that don’t fit together and don’t fit the transformation vision Hiring, developing and promoting people who can implement the change vision Reinvigorating the process with new projects, themes and change agents
Several of the respondents talked about how they were aware of the changes leading to improvements at all levels, that people take great pride in their accomplishments, and they want to help future development teams continue these improvements. The mangers were especially grateful for the shift from random to total utilization review of every patient encounter. They saw that as the best means of ensuring the mission to provide the best preventative care for their patients. Each of the respondents made it clear that the only thing that they can count on in the future is change, which was attributed to a frequent statement by the CEO.
**8. Anchoring new approaches in the culture** Creating better performance through customer productivity-oriented behavior, more and better leadership, and more effective management Articulating the connections between new behaviors and organizational success Developing means to ensure leadership development and succession
The COO notes that hiring new associate dentists’ right out of dental schools that use the DDS diagnostic coding terminology has made a significant difference in immediate productivity for the new associate and the practice group, as well as for smooth integration of the newly hired dentist into the practice culture. One respondent described that after the “big bang” and all the work that went into the adoption of the EHR, the idea of thinking about new approaches is “part of our fiber.” Evidence of the culture shift is the degree to which the CEO, who often dominated larger meetings as the driving force of change, is now less directive in meetings, knowing that others are now leading the charge and ensuring the use of the EHR and diagnostic codes.

**Table 2:** Number of files reviewed as function of type and version

Type	Pre-implementation	Post-implementation	Total
Forms	11	7	18
Policies	40	38	78
Total	51	45	96

**Table 3:** Number of files reviewed as function of types and versions

	Pre-implementation Version	Post-implementation Version	Pre- and Post-implementation Versions remained the same	Updated files	# of files updated to contain “diagnosis”, or description of a diagnosis
Forms	11	7	2	1	0
Policies	40	38	37	30	2
Total	51	45	39	31	2

**Note:** Implementation refers to the implementation of the DDS terminology in the practice

**Table 4:** Number of files as function of types and word “diagnosis”, description of diagnosis, and diagnostic reference

	Forms (n=11) (Pre-implementation version)		Forms (n=7) (Post-implementation version)		Policies (n=40) (Pre-implementation version)		Policies (n=38) (Post-implementation version)	
	Yes (%)	No (%)	Yes (%)	No (%)	Yes (%)	No (%)	Yes (%)	No (%)
Word “diagnosis”	2 (18)	9 (82)	9 (14)	6 (86)	16 (40)	24 (60)	15 (39)	23 (61)
Description of diagnosis	7 (64)	4 (36)	4 (57)	3 (43)	28 (70)	12 (30)	27 (71)	11 (29)
Specific diagnostic term	0 (0)	11 (100)	0 (0)	7 (100)	0 (0)	40 (100)	0 (0)	38 (100)

**Note:** Number in parenthesized notes frequencies. Implementation refers to the implementation of the DDS terminology in the practice. “Diagnosis” includes the variants described in the methods. Number in parenthesis denotes frequencies.
